# 5-Methylcytosine RNA Methyltransferases-Related Long Non-coding RNA to Develop and Validate Biochemical Recurrence Signature in Prostate Cancer

**DOI:** 10.3389/fmolb.2021.775304

**Published:** 2021-12-01

**Authors:** Ke Wang, Weibo Zhong, Zining Long, Yufei Guo, Chuanfan Zhong, Taowei Yang, Shuo Wang, Houhua Lai, Jianming Lu, Pengxiang Zheng, Xiangming Mao

**Affiliations:** ^1^ Department of Urology, Zhujiang Hospital, Southern Medical University, Guangzhou, China; ^2^ Department of Urology, The Hospital of Trade-Business in Hunan Province, Changsha, China; ^3^ Department of Urology, Fuqing City Hospital Affiliated with Fujian Medical University, Fuzhou, China

**Keywords:** 5-methylcytosine in RNA (m5C), lncRNA, biochemical recurrence, prostate cancer, prognostic model

## Abstract

The effects of 5-methylcytosine in RNA (m5C) in various human cancers have been increasingly studied recently; however, the m5C regulator signature in prostate cancer (PCa) has not been well established yet. In this study, we identified and characterized a series of m5C-related long non-coding RNAs (lncRNAs) in PCa. Univariate Cox regression analysis and least absolute shrinkage and selector operation (LASSO) regression analysis were implemented to construct a m5C-related lncRNA prognostic signature. Consequently, a prognostic m5C-lnc model was established, including 17 lncRNAs: *MAFG-AS1*, *AC012510.1*, *AC012065.3*, *AL117332.1*, *AC132192.2*, *AP001160.2*, *AC129510.1*, *AC084018.2*, *UBXN10-AS1*, *AC138956.2*, *ZNF32-AS2*, *AC017100.1*, *AC004943.2*, *SP2-AS1*, *Z93930.2*, *AP001486.2*, and *LINC01135*. The high m5C-lnc score calculated by the model significantly relates to poor biochemical recurrence (BCR)-free survival (*p* < 0.0001). Receiver operating characteristic (ROC) curves and a decision curve analysis (DCA) further validated the accuracy of the prognostic model. Subsequently, a predictive nomogram combining the prognostic model with clinical features was created, and it exhibited promising predictive efficacy for BCR risk stratification. Next, the competing endogenous RNA (ceRNA) network and lncRNA–protein interaction network were established to explore the potential functions of these 17 lncRNAs mechanically. In addition, functional enrichment analysis revealed that these lncRNAs are involved in many cellular metabolic pathways. Lastly, *M* *AFG-AS1* was selected for experimental validation; it was upregulated in PCa and probably promoted PCa proliferation and invasion *in vitro*. These results offer some insights into the m5C's effects on PCa and reveal a predictive model with the potential clinical value to improve the prognosis of patients with PCa.

## Introduction

Prostate cancer (PCa) ranks as the second most commonly diagnosed tumor and the fifth leading cause of mortality in men worldwide, with an increasing trend in incidence (Sung et al., 2021). Moreover, it is reported that the estimated new cases of PCa for 2021 in the United States will probably hit 248,530 with its estimated deaths up to about 34,130, leading this common male malignancy to be a considerable challenge and economic burden to both health services and society (Siegel et al., 2021).

Thankfully, 5-year relative survival rates between localized PCa and metastatic one could differ; the 5-year relative survival rate of localized PCa is >99%, but when it comes to distant/metastatic PCa, the unfavorable 5-year relative survival rate decreases to about 30.6% (National Cancer Institute, 2021), suggesting that metastasis mainly accounts for poor prognoses of patients. Collectively, clinical predicaments that PCa has brought include distinguishing whether a PSA (prostatic specific antigen) detection-based localized PCa presents as an indolent or aggressive one, determining the optimal collocation sequence of systemic therapies for both metastatic castration-sensitive and castration-resistant PCa (mCSPC and mCRPC), and developing promising biomarkers to guide treatment options and improve the prognoses of patients with metastatic PCa (Ku et al., 2019).

High heterogeneity is commonly considered to be one of the significant hallmarks of PCa. The high heterogeneity, mainly characterized by multiple genomic alterations, contributes to cancer initiation, progression and metastasis, and difficulty for the diagnosis, prognosis, and treatment (Dinescu et al., 2019). In addition to genomic aberrations, epigenetic modifications that recently have risen to fame have been reported to be associated with cancer progression and might provide new insights to innovate novel diagnostic and therapeutic strategies. For decades, epigenetics has been prominently acquainted with various and reversible chemical modifications of DNA and histones influencing gene expression while bypassing genomic alteration at the same time. However, in recent years, another layer of gene regulation at the RNA level, known as epitranscriptomics (Deng et al., 2018), has attracted increasing attention and interest in the scientific community with advanced molecular technologies emerging. Compared with the relatively limited spectrum of DNA modifications (six kinds), the abundance of modifications in RNA presents much higher, with over 170 types identified so far. Generally, 5-methylcytosine in DNA (5mC) and its oxidized derivatives, including 5-hydroxymethylcytosine (5hmC), 5-formylcytosine (5fC), and as 5-carboxylcytosine (5caC) are the most well-documented ones at the DNA level (Bohnsack et al., 2019), functioning epigenetically as gene expression regulators via plenty of diverse mechanisms. However, 5-methylcytosine is also detected in several RNA species (m5C), emerging as a critical modulator in many aspects of gene expression, including RNA export, ribosome assembly, translation, and RNA stability (Bohnsack et al., 2019; Dou et al., 2020; Rong et al., 2021).

Long non-coding RNAs (lncRNAs), one category of the non-coding RNA family, have gained intensive attention ever since they were re-classified as critical regulators of gene expression at both transcriptional and post-transcriptional levels rather than useless non-coding materials, functioning in multiple fundamental cellular processes, even in cancer states. Diverse functions, along with the large abundance of lncRNAs, indicate a promising future for lncRNAs to act as both promotors and suppressors of tumor, and accordingly, cumulative evidence has significantly emerged greatly in the past few years (Liang et al., 2019; Hong et al., 2020; Goodall and Wickramasinghe, 2021). For example, the lncRNA *HOTAIR* has been found highly expressed in primary breast tumors and metastases, promoting cancer progression (Gupta et al., 2010), while Second Chromosome Locus Associated with Prostate-1, *LINC00913*, tends to be a promising biomarker given that its expression level is independently related to poor prognosis (Prensner et al., 2013). In our previous study, *SNHG1* has also been proved to be a novel lncRNA, contributing to the malignant progression in PCa (Tan et al., 2021). Previously, methylation of 5-cytosine at the post-transcriptional level was found only in tRNAs and rRNAs. Still, in the era of high-throughput sequencing, m5C is now validated to widely exist in coding RNAs plus other noncoding RNAs such as lncRNAs, microRNAs, etc. Despite accumulating studies focusing on m5C in mRNA, the role of m5C in lncRNA remains rarely elucidated.

Since the mid-2000s, the sequencing technologies named next-generation sequencing (NGS) have continued to evolve, making great strides in the speed and cost of sequencing cancer (Mardis, 2013; Roychowdhury and Chinnaiyan, 2016). In parallel, the volumes of biological data generated by these high-throughput sequencing technologies are so vast that bioinformatics analysis and high-performance computing are necessarily introduced to data processing (Singer et al., 2019). Thus, more and more researchers utilize bioinformatics analysis to construct models related to diagnosis, treatment, and prognosis of cancer.

In this study, we processed the dataset from The Cancer Genome Atlas (TCGA) database via utilizing the least absolute shrinkage and selector operation (LASSO) algorithm (Tibshirani, 1996) to construct a prognostic model with a m5C-related lncRNA signature to predict the biochemical recurrence (BCR) status of patients with PCa. We verified its efficacy and authenticity with the GSE54460 dataset from the Gene Expression Omnibus (GEO) database. Then the competing endogenous RNA (ceRNA) network and lncRNA–protein network were also established, followed by functional enrichment analysis for further exploration. Finally, we chose lncRNA *MAFG-AS1* for preliminary experimental validation, and a set of functional enrichment analyses were performed to investigate its potential molecular function.

## Materials and Methods

### Data Processing

TCGA dataset for Prostate Adenocarcinoma (PRAD), including a total of 460 patients with prognostic information, was obtained from PCaDB (Li et al., 2021a; Li et al., 2021b) (http://bioinfo.jialab-ucr.org/PCaDB/), a comprehensive and interactive database for transcriptomes from PCa population cohorts. In addition, the complete clinical data of 460 patients were downloaded from TCGA (https://portal.gdc.cancer.gov/) for subsequent analyses. The GSE54460 dataset was downloaded from the GEO database (http://www.ncbi.nlm.nih.gov/geo/). R software version: 4.1.0, website tool “catRAPID omics v2.0,” and website tool “OmicShare tools” were used for all analyses in the study.

### Identification of m5C-Related lncRNAs

Thirteen m5C-related genes were collected from literature mining (Takai et al., 2014; Archer et al., 2016; Blanco et al., 2016; Yamashita et al., 2017; Li Y. et al., 2018; Cheng et al., 2018; García et al., 2018; Chen et al., 2019; Gao et al., 2019; Janin et al., 2019; Carella et al., 2020; He et al., 2020; Mei et al., 2020; Sato et al., 2020). Then, expression data of the 13 m5C-related genes and all lncRNAs were retrieved from the TCGA dataset. Pearson correlation analysis (Schober et al., 2018) was used to explore the correlation between lncRNAs and the 13 m5C-related genes; the criteria were |Pearson R| > 0.4 and *p* < 0.05, and eventually 678 lncRNAs were filtered out. Next, logistic regression analysis (Tolles and Meurer, 2016) and univariate Cox regression analysis (Cox, 1972) were implemented on these 678 lncRNAs for further screening, and eventually, 102 overlapped lncRNAs with *p*-value < 0.01 were obtained.

### Construction and Verification of the m5C-Related Prognostic Model

We applied the LASSO regression model (R package “glmnet”) to shrink the candidate genes and, meanwhile, to establish the predictive model. Ultimately, 17 lncRNAs and their respective coefficients were calculated, and the minimum criteria chose the penalty parameter (λ). The m5C-lnc score was calculated by the formula: $m5C-\mathrm{lnc Score}=\sum_{i=1}^{N}\left(Coef_{i}\times Expression\;level\;of\;lncRNA_{i}\right)$ where *N* (17) denotes the number of lncRNAs included in the predictive model, *Coef*_*i*_ refers to a specific lncRNA's coefficient, and *Expression level of lncRNA*_*i*_ represents the relative expression level of a specific lncRNA. The TCGA PCa patients were separated into low- and high-score subgroups according to the median score (–1.713). The BCR-free time was compared between the two subgroups via Kaplan–Meier (KM) survival analysis (Kaplan and Meier, 1958) in the “survminer” package. The “survivalROC” package was employed to perform 1-, 3-, and 5-year ROC (receiver operating characteristic) curve analyses (Mandrekar, 2010) for assessing the predictive power of the prognostic signature, and we compared the AUC (area under the ROC curve) of m5C-lnc score with the AUCs of other clinicopathological features to determine its clinical value. A cohort from the GEO database (GSE54460) was included for validation of the predictive model. The m5C-lnc score for each patient in the GSE54460 cohort was also calculated by the same formula above. Likewise, patients were separated into two subgroups—low- and high-score subgroups—for KM analysis. Finally, the two subgroups were then compared to validate the predictive model, too. We constructed the nomogram and calibration plots based on our previous study (Zhong et al., 2021).

### Construction of ceRNA Network and lncRNA–Protein Interaction Network and Functional Enrichment Analysis

The “GDCRNAtools” package was employed to construct the ceRNA network (Salmena et al., 2011), and the website tool “catRAPID omics v2.0” (http://service.tartaglialab.com/page/catrapid_omics2_group) was utilized to construct the lncRNA–protein interaction network. The website tool “OmicShare tools” (https://www.omicshare.com/tools/) was then used to explore the functional enrichments of the prognostic lncRNAs.

### Cell Culture, RNA Extraction, and Real-Time Quantitative PCR

PCa cell lines, DU145 and PC-3, were both obtained from the National Collection of Authenticated Cell Cultures. Both cell lines were cultured in Dulbecco's modified Eagle's medium. Culture media contained 10% fetal bovine serum and 1% double antibiotics (penicillin and streptomycin). All cell lines were cultivated at 37°C and 5% CO_2_. Total RNAs were extracted from PC-3 and DU145 cells using Trizol reagent (15596018, Takara). Total RNAs were then reverse-transcribed into cDNA using TransScript All-in-one First-Strand cDNA Synthesis SuperMix for qPCR (AT341-01, TransGen). RT-qPCR (real-time quantitative PCR) was carried out using the PerfectStart Green (AQ601-02, TransGen) in Applied Biosystems 7500 Real-Time PCR System. Finally, the relative expression of *MAFG-AS1* was calculated based on the internal reference glyceraldehyde 3-phosphate dehydrogenase (*GAPDH*). All experiments were carried out with three replicates. The primers of *MAFG-AS1* and *GAPDH* are listed in Supplementary Table S3.

### RNA Interference and Loss of Function Assays

GenePharm Company synthesized small interfering RNAs (siRNAs) targeting *MAFG-AS1*. RT-qPCR validated the transfection efficiency after the transfection of siRNAs along with siRNA-Mate (GenePharm) for 72 h. CCK-8 (Cell Counting Kit-8, MA0218-5, Meilunbio) viability assay and colony formation assay were applied to examine the proliferative ability of PCa cell lines after knocking down *MAFG-AS1*. Transwell assay was conducted to examine the invasiveness of *MAFG-AS1* downregulated cells. Detailed procedures of the above assays are available in our previous study (Zhong et al., 2021). All experiments were implemented with three triplicates. siRNAs targeting sites in *MAFG-AS1* are shown in Supplementary Table S3. The original scans of the plate colony formation assays and Transwell assays are shown in Supplementary Figure S3.

### Statistical Analyses

All bioinformatics analyses were performed by R software version 4.1.0 (The R Project for Statistical Computing, Vienna, Austria). Pearson correlation analysis was carried out to analyze correlations between m5C-related genes and lncRNAs. The “survival” and “survminer” packages were applied for KM plots and Cox regression analysis. GraphPad Prism 7.0 (GraphPad, La Jolla, CA, United States) was utilized to analyze the results of RT-qPCR and cell functional assays. All statistical results were showed as mean ± SD (standard deviation) with two-sided test, and the results with a *p*-value < 0.05 were regarded as statistically significant ones.

## Results

### Identification of m5C-Related lncRNAs With Significant Prognostic Value

**FIGURE 1:**
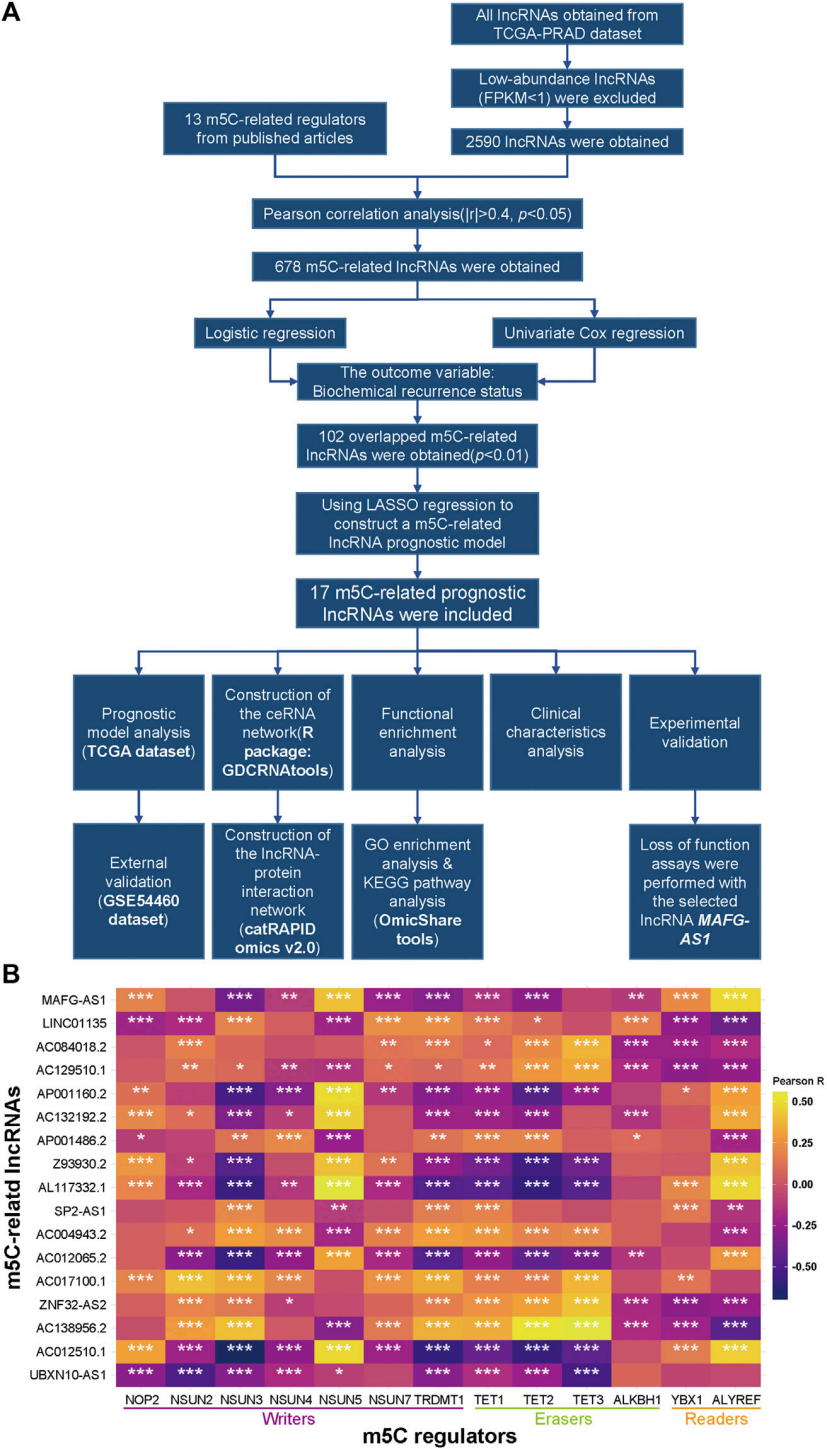
Identification of m5C-related lncRNAs in TCGA-PRAD. **(A)** Flow chart. **(B)** Heatmap of the correlations between m5A-related genes and the 17 prognostic m5C-related lncRNAs. *p < 0.05, **p < 0.01, and ***p < 0.001.

The workflow chart is presented in Figure 1A. Firstly, we searched and identified 13 m5C- regulators from published articles, and then TCGA and GEO datasets of prostate adenocarcinoma were downloaded for further analysis. After extracting the expression matrixes of these m5C regulators and all lncRNAs in the TCGA dataset and excluding lncRNAs with FPKM < 1 (Fragments Per kilobase Million), we performed a Pearson correlation analysis between these m5C-related genes and 2,590 lncRNAs to determine whether a lncRNA was correlated with the m5C modification (|Pearson R| > 0.4 and *p* < 0.05), and consequently we included 678 qualified lncRNAs into logistic regression analysis and univariate Cox regression analysis for further screening (Supplementary Tables S1 and S2). Subsequently, 102 lncRNAs, whose *p*-values were both less than 0.01 in the two analyses mentioned above, were chosen for a LASSO regression analysis to build a m5C-related prognostic model to forecast the BCR in PCa (Supplementary Figure S1A). Eventually, we constructed a model that contains 17 m5C-related lncRNAs (Supplementary Figure S1B), and the correlations between the 17 lncRNAs and the 13 m5C regulators in the TCGA dataset are shown in Figure 1B.

### Clinical Characteristics of the 17 m5C-Related lncRNAs With Prognostic Significance

**FIGURE 2:**
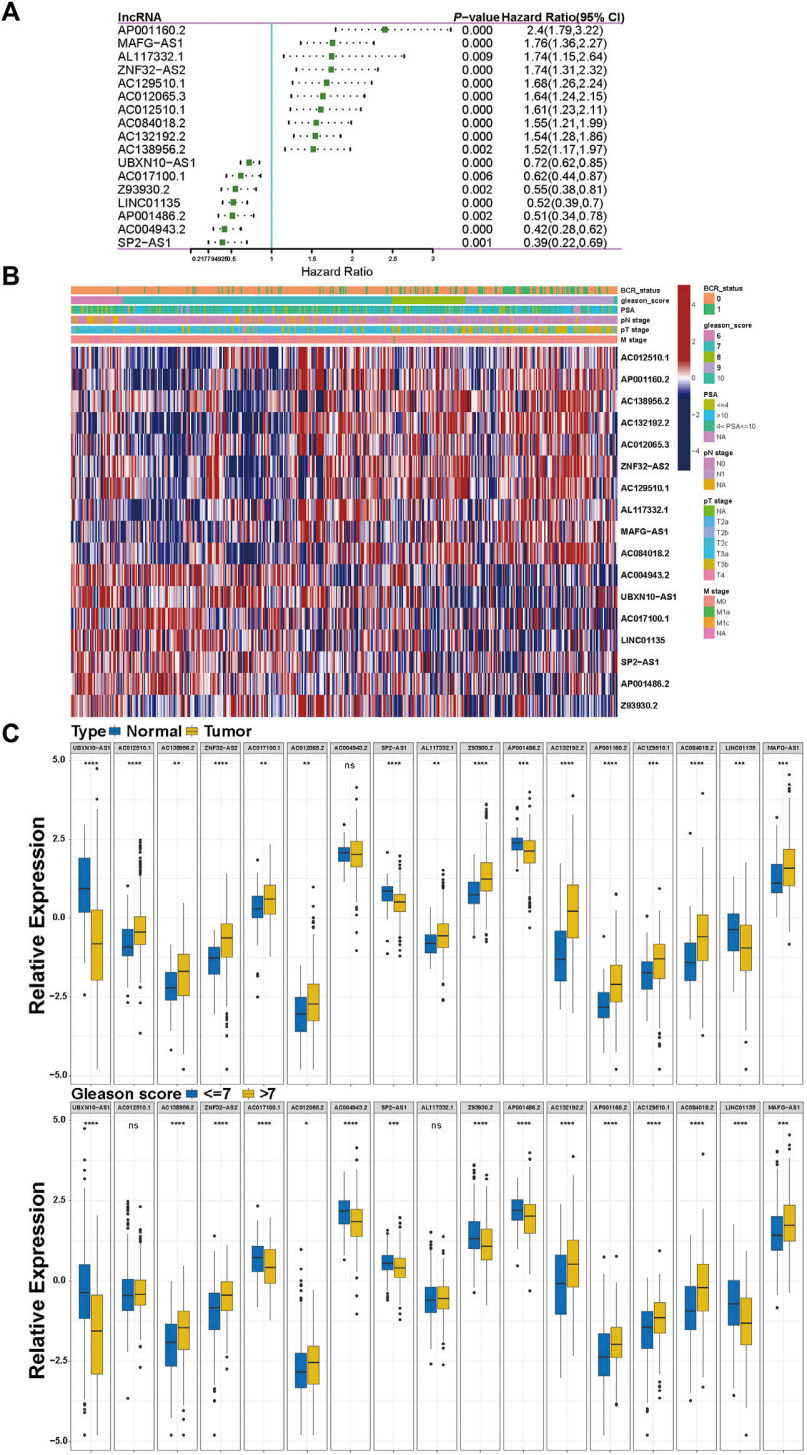
Clinical characteristics of the 17 prognostic m5C-related lncRNAs. **(A)** The result of univariate Cox regression for the 17 prognostic m5C-related lncRNAs. **(B)** The heatmap of clinical signature of the 17 prognostic m5C-related lncRNAs. **(C)** The correlations between the 17 prognostic m5C-related lncRNAs and clinical features. *p < 0.05, **p < 0.01, ***p < 0.001, ****p < 0.0001.

Given the LASSO regression analysis, we focused on these 17 lncRNAs' clinical value. The univariate Cox analysis results of the 17 lncRNAs are shown in Figure 2A; 7 lncRNAs (*UBXN10-AS1*, *AC017100.1*, *Z93930.2*, *LINC01135*, *AP001486.2*, *AC004943.2*, and *SP2-AS1*) tend to be protective factors with HR (hazard ratio) < 1, while another 10 lncRNAs' HRs are more than 1 indicating their high-risk relation with the BCR of PCa. We also separated patients into high-expression and low-expression subgroups based on the median of each lncRNA's expression value. We performed KM survival analysis to detect a significant difference in BCR status between two subgroups. As a result, all 17 KM survival analyses revealed that the high-expression subgroups belonging to the 7 protective lncRNAs mentioned above harbored favorable BCR status. In contrast, the high-expression subgroups of another 10 high-risk lncRNAs gained poorer BCR status, and meanwhile, all the KM analyses hit a *p*-value < 0.05, as shown in Supplementary Figure S2. Then we extracted 460 patients' clinical data, including BCR status, Gleason score (GS), PSA, pathological T stage, pathological N stage, and metastatic stage from the TCGA-PRAD dataset to inspect the 17 lncRNAs' signatures, and the heatmap is displayed in Figure 2B. Moreover, we implemented a further analysis to explore the correlation between 17 m5C-related modulators and clinical features after classifying these patients into several subgroups, as shown in Figure 2C and Supplementary Figure S1E. The expression of all lncRNAs except *AC004943.2* in tumors exhibited significance, compared with that in adjacent normal tissue. Regarding the T stage, N stage, and GS subgroups, five, four, and two lncRNAs did not show significant difference, respectively. Consequently, most of these lncRNAs revealed their predictive value.

### Construction and Validation of the m5C-Related lncRNA Prognostic Model

**FIGURE 3:**
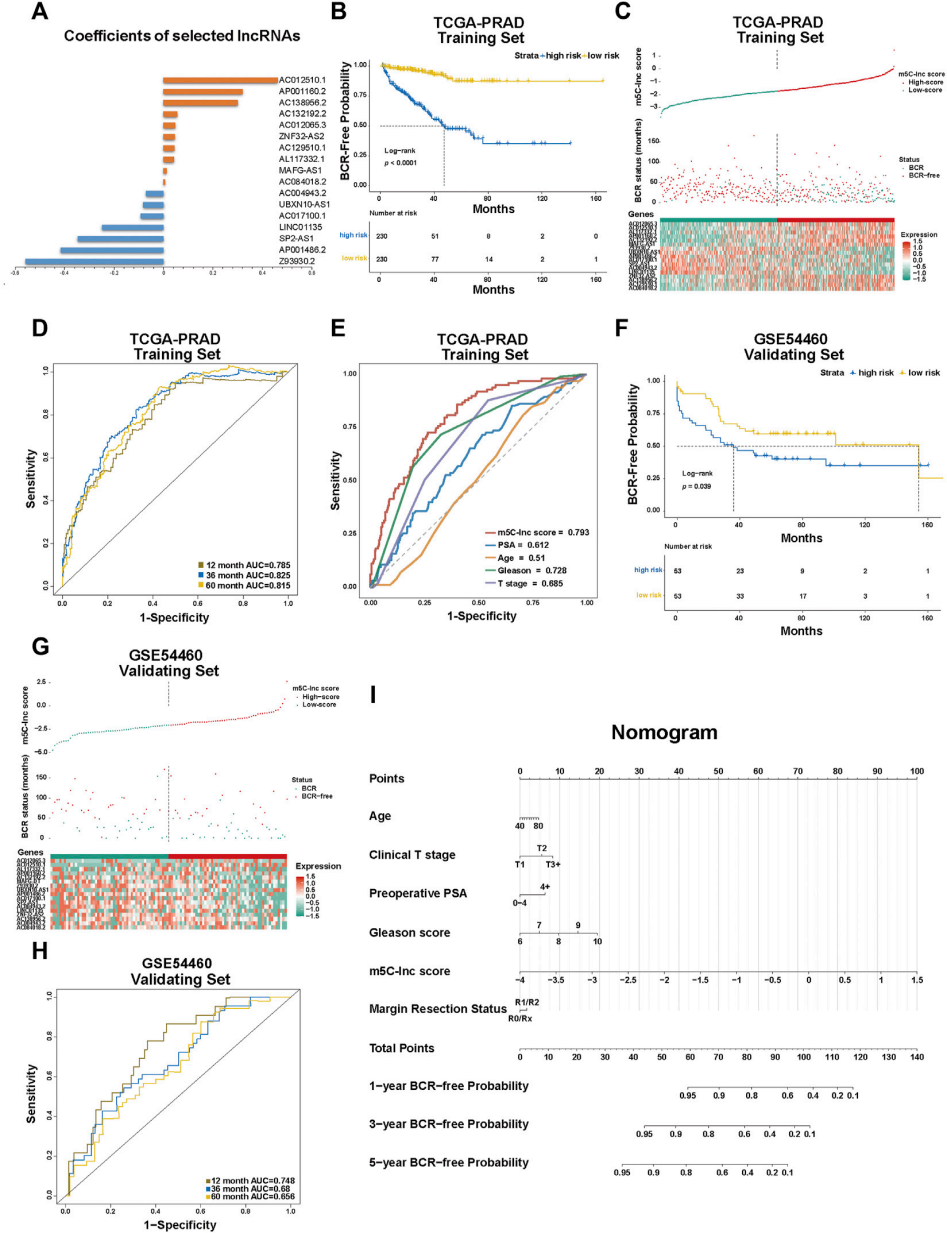
Construction and validation of the m5C-Related lncRNA prognostic model in patients with prostate cancer. **(A)** The coefficients of the prognostic 17 m5C-related lncRNAs calculated by LASSO regression analysis. **(B)** Kaplan–Meier survival analysis shows that BCR-free time of patients with high m5C-lnc scores is significantly shorter than of those with low m5C-lnc scores. **(C)** The separation of low and high m5C-lnc scores in patients with PCa; scatter plot depicts the distribution of BCR status between two groups divided by the median m5C-lnc score; the heatmap shows that patients with shorter BCR-free time displayed higher gene expression level of risk factors (*AP001160.2*, *MAFG-AS1*, *AL117332.1*, *ZNF32-AS2*, *AC129510.1*, *AC012065.3*, *AC012510.1*, *AC084018.2*, *AC132192.2*, and *AC138956.2*), while patients with longer BCR-free time displayed higher gene expression level of protective factors (*UBXN10-AS1*, *AC017100.1*, *AC004943.2*, *SP2-AS1*, *Z93930.2*, *AP001486.2*, and *LINC01135*). **(D)** ROC curves illustrated the sensitivities of the prognostic m5C-lnc score model predicting 1-, 3- and 5-year BCR status. **(E)** ROC sensitivity comparison between m5C-lnc score and other clinical features including PSA level, age, Gleason score, and T stage. **(F–H)** The validation for the constructed prognostic model using GSE54460 dataset. **(I)** The nomogram containing the m5C-lnc score and other clinicopathological features for prognosis prediction.

Following the analyses above, we then constructed the m5C-related lncRNA prognostic model named m5C-lnc score with these 17 lncRNAs, and their coefficients calculated by LASSO regression analysis are shown in Figure 3A. Patients in the TCGA cohort were divided into low-score and high-score subgroups based on the m5C-lnc score attained by the formula above and its median. KM survival curves demonstrated that patients in the low-score subgroup had better clinical outcomes (longer BCR-free time), as shown in Figure 3B. m5C-lnc score and BCR status distribution of TCGA-PRAD dataset are depicted in Figure 3C. Subsequently, ROC analysis showed that the predictive m5C-lnc score model whose 1-, 3-, and 5-year AUCs were all about 0.8 (12-month AUC = 0.785, 36-month AUC = 0.825, and 60-month AUC = 0.815; Figure 3D) had excellent sensitivity and specificity, exhibiting better power to predict BCR status in the TCGA cohort; moreover, compared with the AUCs of other prognostic factors such as PSA, age, GS, and pathological T stage (AUC of PSA = 0.612, AUC of age = 0.51, AUC of GS = 0.728, and AUC of pathological T stage = 0.685), the AUC of the m5C-lnc score hit the highest score (0.793; Figure 3E), suggesting the model's better sensitivity, specificity, and clinical predictive value. The decision curve analysis (DCA) of the TCGA cohort indicated that the clinical benefit rate of the m5C-lnc score model was significantly higher than that of other clinical characteristics based on GS and T stage, respectively (Supplementary Figure S1C). Moreover, we performed univariate Cox regression with m5C-lnc score and other clinical pathological features including age, PSA, GS, and pathological T stage, respectively. As a result, all factors except age were significantly associated with patients' BCR status, as shown in Supplementary Figure S4A; PSA, GS, m5C-lnc score, and pathological T stage were then included in further multivariate Cox regression, and consequently, m5C-lnc score presented as the primary risk factor with the highest HR of about 3, as shown in Supplementary Figure S4B.

To validate the predictive value of the established m5C-related lncRNA signature, we applied the GSE54460 dataset to verify the results; likewise, patients in the GSE54460 dataset were also separated into low-score and high-score groups by the median m5C-lnc score. Consequently, the survival analysis results were similar to those in the TCGA dataset: patients with higher m5C-lnc scores had a lower BCR-free rate and a shorter BCR-free time (Figure 3F). Likewise, m5C-lnc score and BCR status distribution are illustrated in Figure 3G. The ROC analysis indicated that these m5C-related regulators' signature also had a promising prognostic value for patients in the GSE54460 dataset given that the 1-, 3-, and 5-year AUCs were all around 0.7 (12-month AUC = 0.748, 36-month AUC = 0.68, and 60-month AUC = 0.655; Figure 3H). Nomogram is always used to quantitatively predict the prognosis of patients in clinical practice by calculating the relative points on a set of scales. In our study, we illustrated our predictive model in the form of a nomogram as shown in Figure 3I to predict 1-, 3-, and 5-year BCR-free probabilities, respectively, for patients with PCa according to the “Total Points.” A total point considers six variables including PSA, age, T-stage, GS, m5C-lnc score, and resection status. With the scale bar “Points” to be a reference, the primary value of each variable could correspond to a point on the “Points” scale, and all the six relative points are added together to make the total points. Ultimately, the probabilities of 1-, 3-, or 5-year BCR-free time for each patient could be quantified respectively by the points corresponding to the scale bar “Total Points.” Besides, we also carried out a calibration curve analysis to demonstrate that the estimated 3- and 5-year BCR-free time were in accordance with the actual scenario (Supplementary Figure S1D).

### Construction of the Competing Endogenous RNA Network and the lncRNA–Protein Interaction Network

**FIGURE 4:**
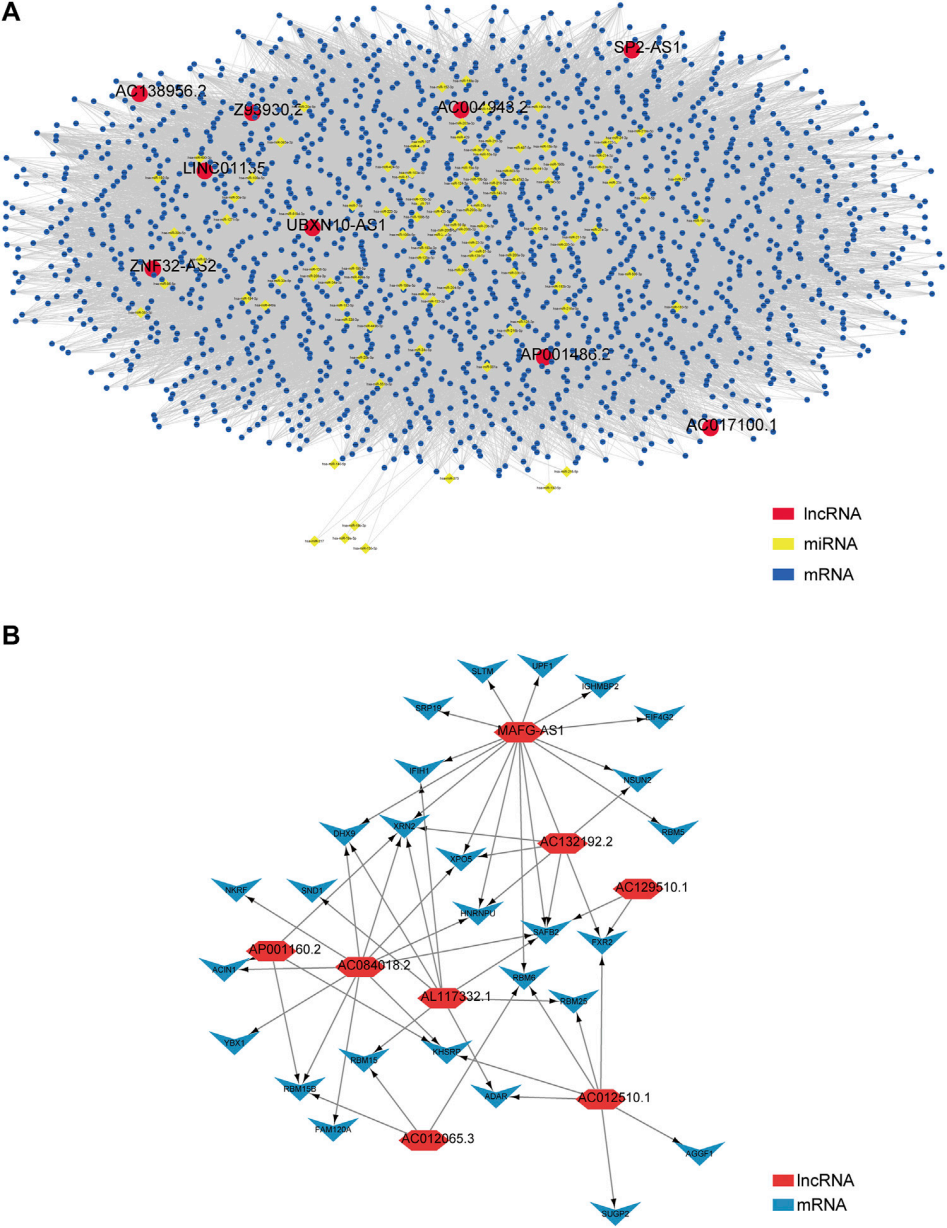
Construction of the ceRNA network and lncRNA–protein network based on the 17 prognostic m5C-related lncRNAs in PCa patients. **(A)** A ceRNA network containing 9 lncRNAs (*UBXN10-AS1*, *AC138956.2*, *ZNF32-AS2*, *AC017100.1*, *AC004943.2*, *SP2-AS1*, *Z93930.2*, *AP001486.2*, and *LINC01135*), 106 miRNAs, and 1710 mRNAs shows their lncRNA–miRNA–mRNA pathways interaction. **(B)** A lncRNA–protein interaction network relates to the rest of 8 lncRNAs (*AC012510.1*, *AC012065.3*, *AL117332.1*, *AC132192.2*, *AP001160.2*, *AC129510.1*, *AC084018.2*, and *MAFG-AS1*).

Next, we explored how these lncRNAs function in biological processes by using the R package called “GDCRNAtools” to construct a ceRNA network and visualizing it with the help of the software Cytoscape (Shannon et al., 2003). Consequently, 9 lncRNAs (*UBXN10-AS1*, *AC138956.2*, *ZNF32-AS2*, *AC017100.1*, *AC004943.2*, *SP2-AS1*, *Z93930.2*, *AP001486.2*, and *LINC01135*), 106 miRNAs, and 1,710 mRNAs were included in the ceRNA network with their lncRNA–miRNA–mRNA pathways interacting mutually, as shown in Figure 4A. For the rest of lncRNAs (*AC012510.1*, *AC012065.3*, *AL117332.1*, *AC132192.2*, *AP001160.2*, *AC129510.1*, *AC084018.2*, and *MAFG-AS1*), we utilized an online website tool called catRAPID omics v2.0 (Agostini et al., 2013) to inspect the lncRNA–protein interaction. After inputting each lncRNA's information, the tool automatically predicted the candidate proteins and presented the result list. Given the build-in ranking system (0 star to 3 stars), we selected 27 candidate proteins with 2.5 and more stars to construct the interaction network. Likewise, the lncRNA–protein interaction network was visualized by Cytoscape, as shown in Figure 4B. Detailed data can be found in Supplementary Tables S4 and S5.

### Functional Enrichment Analysis of 17 m5C-Related lncRNAs With Prognostic Value

**FIGURE 5:**
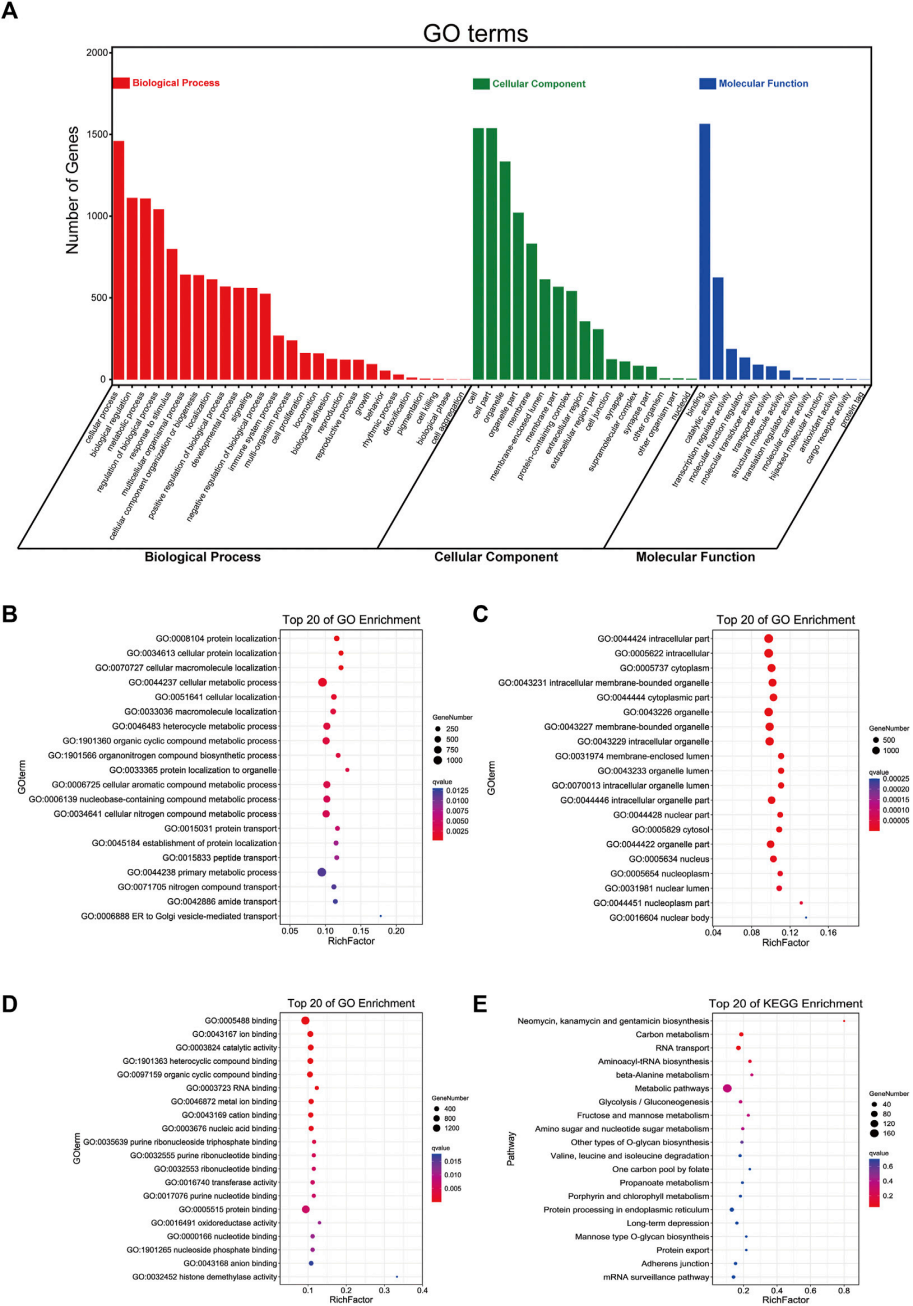
Functional enrichment analysis of 17 m5C-related lncRNAs with prognostic value. **(A)** The summary bar plot of GO enrichments. **(B–D)** The respective top 20 GO enrichments of biological process, cellular component, and molecular function. **(E)** The top 20 KEGG pathways.

Furthermore, we used these 17 lncRNAs to perform functional enrichment analysis with an online tool called OmicShare tools. The summary of GO (Gene Ontology) enrichment and details about its three parts are shown in Figures 5A–D, and the KEGG (Kyoto Encyclopedia of Genes and Genomes) pathway enrichment is also demonstrated in Figure 5E. Accordingly, we found that the top five GO Biological Processes that these genes were primarily enriched in are heterocycle metabolic process, nucleobase-containing compound metabolic process, organic cyclic compound metabolic process, cellular nitrogen compound metabolic process, and cellular aromatic compound metabolic process (Figure 5B); neomycin, kanamycin, and gentamicin biosynthesis, carbon metabolism, and RNA transport were significantly enriched in KEGG Pathway (Figure 5E). These data may bring us some insights into the potential functions of these m5C-related lncRNAs in PCa. Complete information are shown in Supplementary Table S6.

### Selecting *MAFG-AS1* for Experimental Validation and Individual Analysis

**FIGURE 6:**
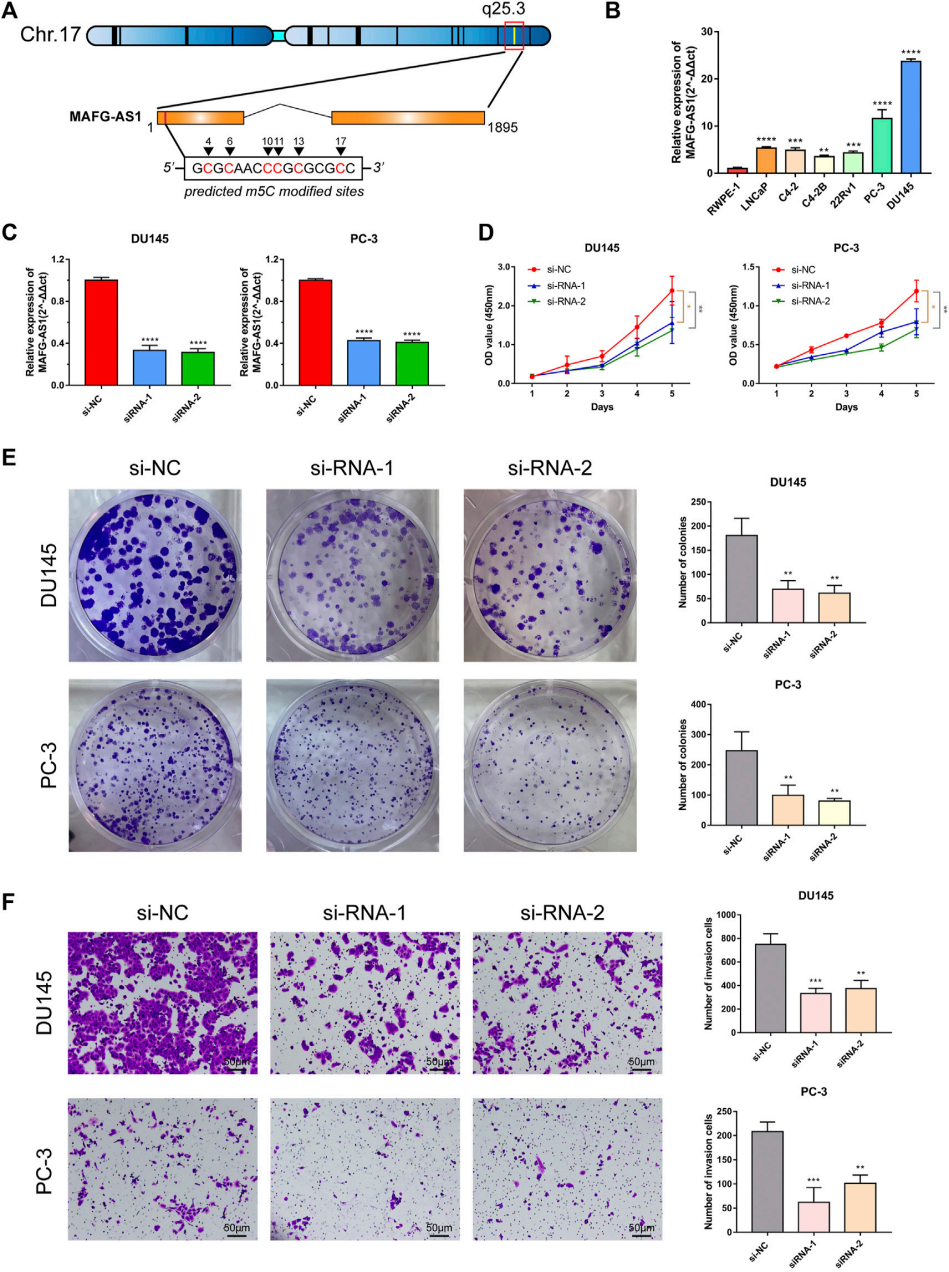
Characteristics of MAFG-AS1 and its functional effect on PCa. **(A)** The basic information of *MAFG-AS1* and its m5C sites predicted by the website tool, iRNAm5C-PseDNC. **(B)** Basal expression level of *MAFG-AS1* in PCa cell lines. **(C)** RT-PCR verified the transfection efficiency of siRNAs targeting to *MAFG-AS1* in PC-3 and DU145. **(D)** CCK-8 assay revealed that knockdown of *MAFG-AS1* inhibited PCa cells viability. **(E)** Knockdown of *MAFG-AS1* inhibited colony formation in PCa cells. **(F)** Knockdown of *MAFG-AS1* attenuated PCa cells' invasive ability. Error bar indicates mean ± SD. *p < 0.05, **p < 0.01, and ***p < 0.001, ****p < 0.0001.

With the univariate Cox regression analysis mentioned above, 10 lncRNAs' HRs are greater than 1, indicating their potential promoting PCa. Among them, the abundance of *MAFG-AS1* is the highest (Figure 2C), and its HR ranks second (Figure 2A). Therefore, we chose to implement the experimental validation with *MAFG-AS1*. Based on the data provided on LNCipedia (Volders et al., 2019), a website with comprehensive information of human lncRNAs, *MAFG-AS1*, known as *MAFG-DT*, is located at q25.3 of chromosome 17 with a transcript size of 1,895 bp. Subsequently, we used an online tool called iRNAm5C-PseDNC to identify 5-methylcytosine modified sites of *MAFG-AS1*, and the predicted sites included positions 4, 6, 10, 11, 13, and 17, as shown in Figure 6A.

Firstly, we detected the basal expression level of *MAFG-AS1* in PCa cell lines by RT-qPCR, and it was found that *MAFG-AS1* was highly expressed in all PCa cell lines with the benign prostate cell line RWPE1 to be the reference (Figure 6B). The expressions of *MAFG-AS1* in PC-3 and DU145 cell lines are much higher than that of others, so we chose these two cell lines to construct the *MAFG-AS1* knock-down phenotypes with two siRNAs, respectively. The results of RT-qPCR confirmed that both siRNA fragments significantly silenced the expression of *MAFG-AS1* in PC-3 cell line and DU145 cell line (Figure 6C). After examining the transfection efficiency in PC-3 and DU145 cell lines, we performed a set of assays to verify this specific gene's function on cancer cells. CCK-8 assay and plate colony formation assay suggested that down-regulated *MAFG-AS1* significantly inhibited PCa proliferation ability (Figures 6D, E). Furthermore, knockdown of *MAFG-AS1* attenuated PCa cell's invasion ability via transwell assay (Figure 6F). These results indicated that *MAFG-AS1* acts as a high-risk predictive factor in PCa, and somehow its high expression promotes cancer progression.

**FIGURE 7:**
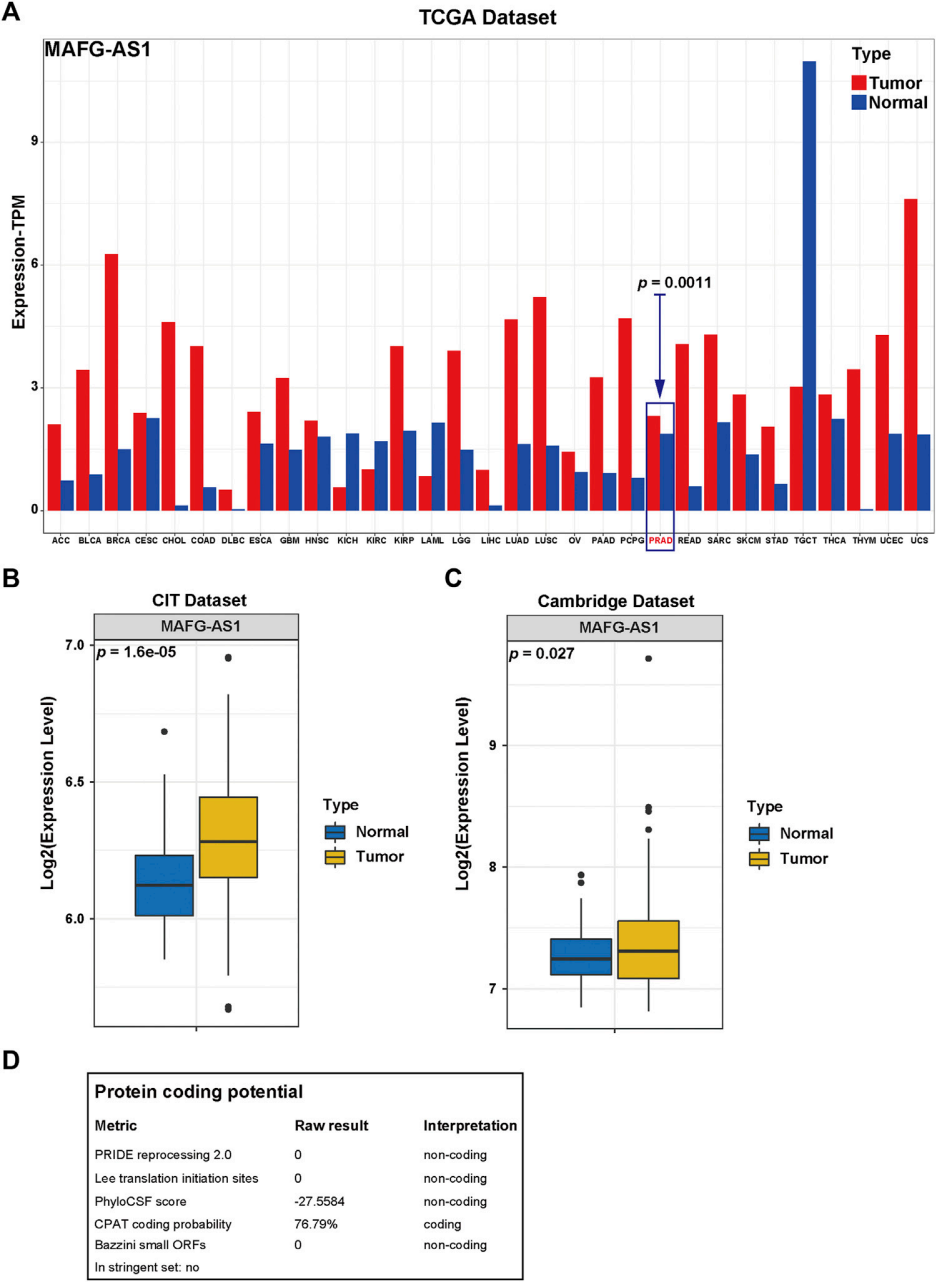
Functional enrichment analysis of MAFG-AS1. **(A)** The pan-cancer expression profile of *MAFG-AS1* provided by the website tool, GEPIA2. **(B, C)** The *MAFG-AS1*'s expression profile in tumor tissue and adjacent normal tissue in two datasets, CIT dataset and Cambridge dataset. **(D)** The protein coding potential of *MAFG-AS1* predicted by the website tool, LNCipedia.

After determining *MAFG-AS1*'s promotion in PCa's proliferation and invasion, we attempted to inspect its molecular function further. First of all, we checked out *MAFG-AS1*'s expression profile with a pan-cancer analysis on GEPIA 2 (Tang et al., 2017) as shown in Figure 7A, and it turned out that *MAFG-AS1* is highly expressed in most cancers. Additionally, we detected a significant difference in the expression of *MAFG-AS1* in tumor tissue and adjacent normal tissue in another two datasets, CIT dataset and Cambridge Dataset (GSE70768), as shown in Figures 7B, C; the expression of *MAFG-AS1* is higher in tumor tissue than in the adjacent normal tissue. Meanwhile, we also explored the relation between *MAFG-AS1*'s expression and clinical characteristics in patients with PCa. It was found that patients with high GS tended to have high *MAFG-AS1* expression level (GS = 6: 1.29 ± 0.70, GS = 7: 1.58 ± 0.84, and GS ≥ 8: 1.84 ± 0.92, *p* < 0.001), and the significant difference was also observed in the pathological T stage category, lymph node metastasis category, and BCR status category (≤T2c: 1.52 ± 0.81, T3/T4: 1.75 ± 0.92, *p* = 0.005; lymph node metastasis-positive: 1.89 ± 0.99, lymph node metastasis-negative: 1.62 ± 0.87, *p* = 0.018; BCR-positive: 1.94 ± 0.91, BCR-negative: 1.58 ± 0.86, *p* < 0.001), as shown in Supplementary Table S8. Although it was not included in the ceRNA network (Figure 4A), it interacts with 15 proteins in the lncRNA–protein network, ranking first, which indicates it might play an essential role in cancer progression. Then we implemented Spearman's correlation analysis between *MAFG-AS1* and all other genes in TCGA-PRAD dataset to screen out the correlated genes of *MAFG-AS1* for further functional enrichment analysis. The criteria were |Spearman R| > 0.4 as well as *p*-value < 0.05, and 833 correlated genes were found. Next, we utilized these genes to perform functional enrichment analysis via the website tool OmicSharetools. Consequently, most genes were enriched in these terms: RNA processing, mRNA metabolic process, ribonucleoprotein complex biogenesis, protein targeting, and mRNA catabolic process (GO Biological Process) and ribosome, spliceosome, and RNA transport (KEGG Pathway) (Supplementary Figures S5A–E). Complete functional enrichment documents can be found in Supplementary Table S7. These results suggested that *MAFG-AS1* might influence cancer development by interacting with proteins to interrupt the activities of RNAs, especially mRNAs. Lastly, we used LNCipedia to predict *MAFG-AS1*'s protein-coding potential. The website would carry out its prediction with various applications such as CPC, HMMER, PRIDE, PhyloCSF, CPAT, and Ribosome-profiling. As a result, only CPAT provided a coding probability of 76.79% (Figure 7D). It seems that *MAFG-AS1* encodes protein with little potential.

## Discussion

In the United States, PCa's incidence has already ranked first in males, with its estimated death cases ranking second. About 35% of patients will go through BCR after radical prostatectomy within a decade (Freedland et al., 2005). It could heavily threaten patients' quality of life and even lifespan when it comes to BCR because patients with BCR have higher chances of developing metastatic PCa. Thus, risk stratification of patients via PSA, GS, and pathological stage, etc., becomes significant for prognosis prediction (Kreuz et al., 2020). However, with a deeper understanding of PCa, they gradually fail to provide more precise prediction. Therefore, novel predictive models are urgent to be discovered and applied in clinical practice.

More recently, with well-improved methods via both analytical chemistry and high-throughput sequencing for detection, RNA modification has come to prominence (Roundtree et al., 2017). And numerous pieces of evidence suggest that dysfunction of RNA epigenetic pathway is strongly associated with human diseases, especially cancer (Barbieri and Kouzarides, 2020). In terms of RNA modifications, apart from the famous N6-methyladenosine (m6A) modification, 5-methylcytosine (m5C) has also gradually attracted great interest with the development of detective techniques; three effective techniques have become dominant: detection at single nucleotide resolution with bisulfite conversion, also known as bisulfite sequencing (bsRNA-seq), 5-azacytidine-mediated RNA immunoprecipitation (Aza-IP), and methylation individual-nucleotide-resolution cross-linking and immunoprecipitation (miCLIP), making precise m5C mapping more possible (Dinescu et al., 2019). The dynamic activities of RNA modifications to adjust RNAs' functions are technically controlled by three elements (Barbieri and Kouzarides, 2020): enzymes that catalyze the modified site to form (“writers”), reading proteins that detect and bind to the modified sites (“readers”), and enzymes that carry out removing the modified base, ensuring the modifying process is reversible (“erasers”). There are two significant groups of m5C writers: seven members from the NOP2/SUN RNA methyltransferase (NSUN) family and DNA methyltransferase-2 (DNMT2), previously considered to relate to DNA methylation. While writers for m5C are now well documented, the existence of m5C erasers are still unclear given that no currently existing protein can fully reverse m5C to cytosine (Rong et al., 2021).

Previous studies showed the Aly/REF export factor (ALYREF) and Y-box binding protein 1 (YBX1) may act as significant readers for m5C, taking part in cancer-related biological processes (Yang et al., 2017; Chen et al., 2019). NSUN2 (NOP2/Sun domain family, member 2), a major m5C-modifying methyltransferase (writer) of RNA, is highly expressed in various tumors such as the esophagus, liver, pancreas, cervix, prostate, kidney, bladder, thyroid, and breast cancers (Okamoto et al., 2012; Huang et al., 2021). Recently, NSUN2 was reported to promote gastric cancer cell proliferation in a m5C-dependent manner (Mei et al., 2020). These researches demonstrated that m5C modification of RNA plays a vital role in cancer.

Notably, lncRNAs take a significant part of non-coding RNAs; increasing research unveiled those various chemical modifications, including m5C, are found in lncRNAs, and their possible involvement in different types of cancer are also reported previously. For instance, m5C modifications in lncRNAs such as XIST (X-inactive specific transcript), TERC (telomerase RNA component), RPPH1 (ribonuclease P RNA component H1), and ANRIL (antisense non-coding RNA in the INK4 locus) may have an association with leukemia and colorectal cancer, PCa, breast cancer, and PCa as well as lung cancer, respectively (Squires et al., 2012; Amort et al., 2013; Baena-Del Valle et al., 2018; Zhao et al., 2018; Pan et al., 2021). Research reported that an m5C-modified H19 lncRNA might enhance tumorigenesis and progression (Sun et al., 2020). However, researches about m5C-modified lncRNAs impacting on PCa are rarely found.

In our study, we firstly collected the m5C-related genes from published articles, and consequently, 13 m5C-related regulators were found. Then we screened out the lncRNAs related to m5C modification in PCa with TCGA dataset for LASSO regression analysis and tried to construct a prognostic model with 17 lncRNAs obtained by the analysis. After checking out the predictive model's clinical characteristics, we verified the model's value, and it turned out to be a better predictor for the prognosis of PCa patients. Subsequently, a series of bioinformatics analyses, including construction of ceRNA network and lncRNA–protein network and functional enrichment analyses, were carried out to explore the lncRNAs' functional profiles. Given functional analysis outcomes, cell metabolic processes may be involved in these lncRNAs, among which cellular nitrogen compound metabolic process enriched the most genes.

Moreover, we noticed *MAFG-AS1* given its highest abundance and second-highest HR among 10 lncRNAs with their HRs > 1. *MAFG-AS1*, known as *MAFG-DT*, is found to get involved in several tumors such as bladder cancer (Xiao et al., 2020), colorectal cancer (Ruan et al., 2020), lung cancer (Sui et al., 2019), liver cancer (Chen et al., 2021), ovarian cancer (Bai et al., 2021), breast cancer (Jia et al., 2021), and pancreatic cancer (Ye et al., 2020). However, the role of *MAFG-AS1* in PCa has not been unveiled yet. Therefore, we picked *MAFG-AS1* for experimental validation. The results revealed that *MAFG-AS1* could promote proliferation and invasion in PCa, indicating a cancer-promoting effect. At the same time, we inspected its expression levels in both tumor tissue and adjacent normal tissue, via bioinformatics analysis, and its relation with various clinical characteristics. As a result, the expression of *MAFG-AS1* is higher in tumor tissue than in adjacent normal tissue; *MAFG-AS1*'s high expression relates to patient's poor clinical status given the analysis mentioned above. Subsequently, we tried to explore *MAFG-AS1*'s molecular function. As shown in previous results, we use the R package “GDCRNAtools” (Li R. et al., 2018) to construct the ceRNA network by putting the expression matrixes of these 17 lncRNAs, all miRNAs and all mRNAs from TCGA dataset, in the “miRcode” database for exploration. Unluckily, *MAFG-AS-1* was not included in the ceRNA network above, but it was found to rank first in the lncRNA–protein network, interacting with 15 proteins, all scored with 2.5 or more stars in the build-in system mentioned above. This indicated that *MAFG-AS1* might impact the cancer progression in other ways instead of ceRNA network in PCa. Then the functional enrichment analysis with the 833 correlated genes of *MAFG-AS1* revealed that most genes were found to get involved in RNA processing. Hence, it is likely that *MAFG-AS1* might influence cancer development via interrupting RNAs' activities like biogenesis or degradation. With LNCipedia to predict *MAFG-AS1*'s protein coding potential, only one application called CPAT provided a coding probability of 76.79%. Seemingly, *MAFG-AS1* encodes a protein with little potential.

Due to lncRNA's broad definition, lncRNA's mechanisms function heterogeneously. Spatially, lncRNA's functions could be divided into two significant parts: the nucleus and the cytoplasm. Three fundamental mechanisms are highlighted in the nucleus: scaffolding and integrating regulatory proteins to form macromolecular complexes, localizing to specific sites on genomic DNA, and organizing three-dimensional nuclear structure (Engreitz et al., 2016). One of the best illuminated examples of lncRNA regulating gene expression in the nucleus is Xist (X inactive specific transcript). In the cytoplasm, lncRNAs mainly regulate gene expression by competing for endogenous RNAs (ceRNAs) to competitively bind miRNAs, thereby indirectly affecting the target mRNAs' expression (Salmena et al., 2011). Based on the definition, a lncRNA usually refers to a transcript with a sequence longer than 200 bp and lacks protein-coding potential (Goodall and Wickramasinghe, 2021). However, with the newly emerging advanced techniques like full-length translating mRNA sequencing and ribosome profiling, the long debate whether non-coding RNAs taking account for 98% of the human genome can encode functional proteins besides short peptides seems to be ended by recent studies. Research surprisingly confirmed a hidden human functional proteome encoded by specific lncRNAs, suggesting that the annotation of lncRNAs used before is outdated in some way (Lu et al., 2019). Therefore, we inspected the coding potential of *MAFG-AS1* even though it does not seem to encode functional proteins according to the result. Additionally, *MAFG-AS1* is not included in our constructed ceRNA network but interacts with several proteins to involve some mechanisms inside the nucleus.

Our current study is just one small step into exploring the valuable predictive model based on RNA m5C modification in PCa. Hence, in-depth validation with a clinical trial is definitely needed to confirm its exact value, and further experimental confirmation is also required to illuminate lncRNA *MAFG-AS1*'s effects on PCa. Despite the above limitations, we believe that our predictive model based on m5C-related lncRNA signatures will offer some valuable information to the current clinical predicament in PCa.

To sum, our study revealed that the m5C-related lncRNA signatures could precisely predict BCR in patients with PCa by m5c-lnc score. Besides, the nomogram integrated with m5C-related lncRNA signatures and other clinicopathological features presented the customized BCR-free probability prediction. *MAFG-AS1* was ultimately chosen for further experimental verification, and it was upregulated in PCa and probably promoted PCa aggression *in vitro*.

## Data Availability

The datasets presented in this study can be found in online repositories. The names of the repository/repositories and accession number(s) can be found in the article/Supplementary Material.

## Abbreviations

AUC, area under the ROC curve; BCR, biochemical recurrence; CCK-8, Cell Counting Kit-8; CeRNA, competing endogenous RNA; CIT, Cartes d’Identité des Tumeurs; DCA, decision curve analysis; FPKM, Fragments Per Kilobase Million; GEO, Gene Expression Omnibus; GO, Gene Ontology; GS, Gleason score; HR, hazard ratio; KEGG, Kyoto Encyclopedia of Genes and Genomes; lncRNA, long non-coding RNA; LASSO, least absolute shrinkage and selector operation; m5C, 5-methylcytosine; m6A, N6-methyladenosine; mCRPC, metastatic castration-resistant prostate cancer; mCSPC, metastatic castration-sensitive prostate cancer; NGS, next-generation sequencing; PCa, prostate cancer; PRAD, Prostate Adenocarcinoma; PSA, prostatic specific antigen; ROC, receiver operating characteristic; RT-qPCR, real-time quantitative PCR; siRNA, small interfering RNA; SD, standard deviation; TCGA, The Cancer Genome Atlas.
