# Supplementary figures and images for: 5-Methylcytosine RNA Methyltransferases-Related Long Non-coding RNA to Develop and Validate Biochemical Recurrence Signature in Prostate Cancer
Source: Front Mol Biosci. 2021 Dec 1;8:775304. doi: 10.3389/fmolb.2021.775304 (PMC8672116; doi:10.3389/fmolb.2021.775304)

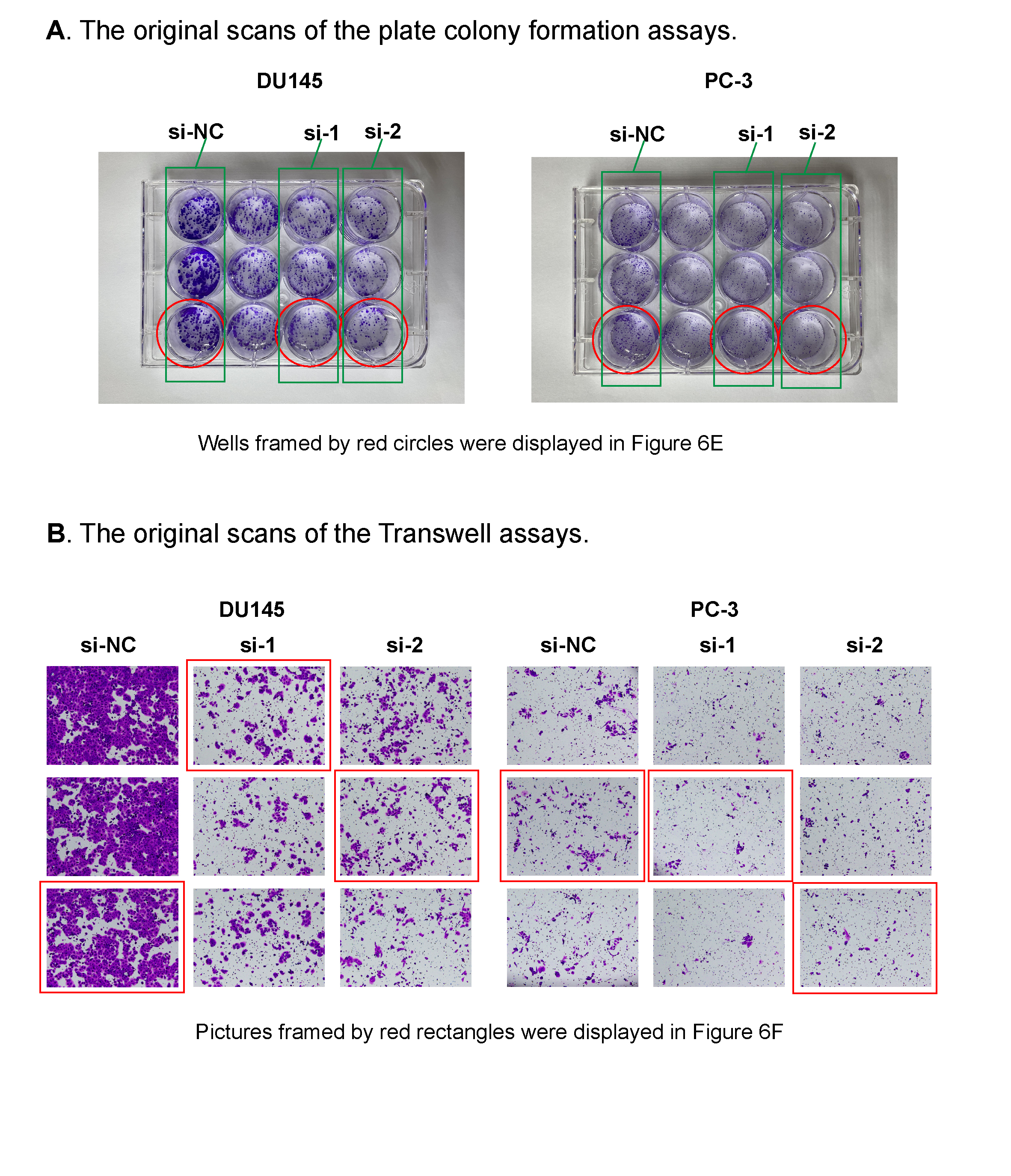

Supplement: Supplementary file 2 [file Image3.JPEG]

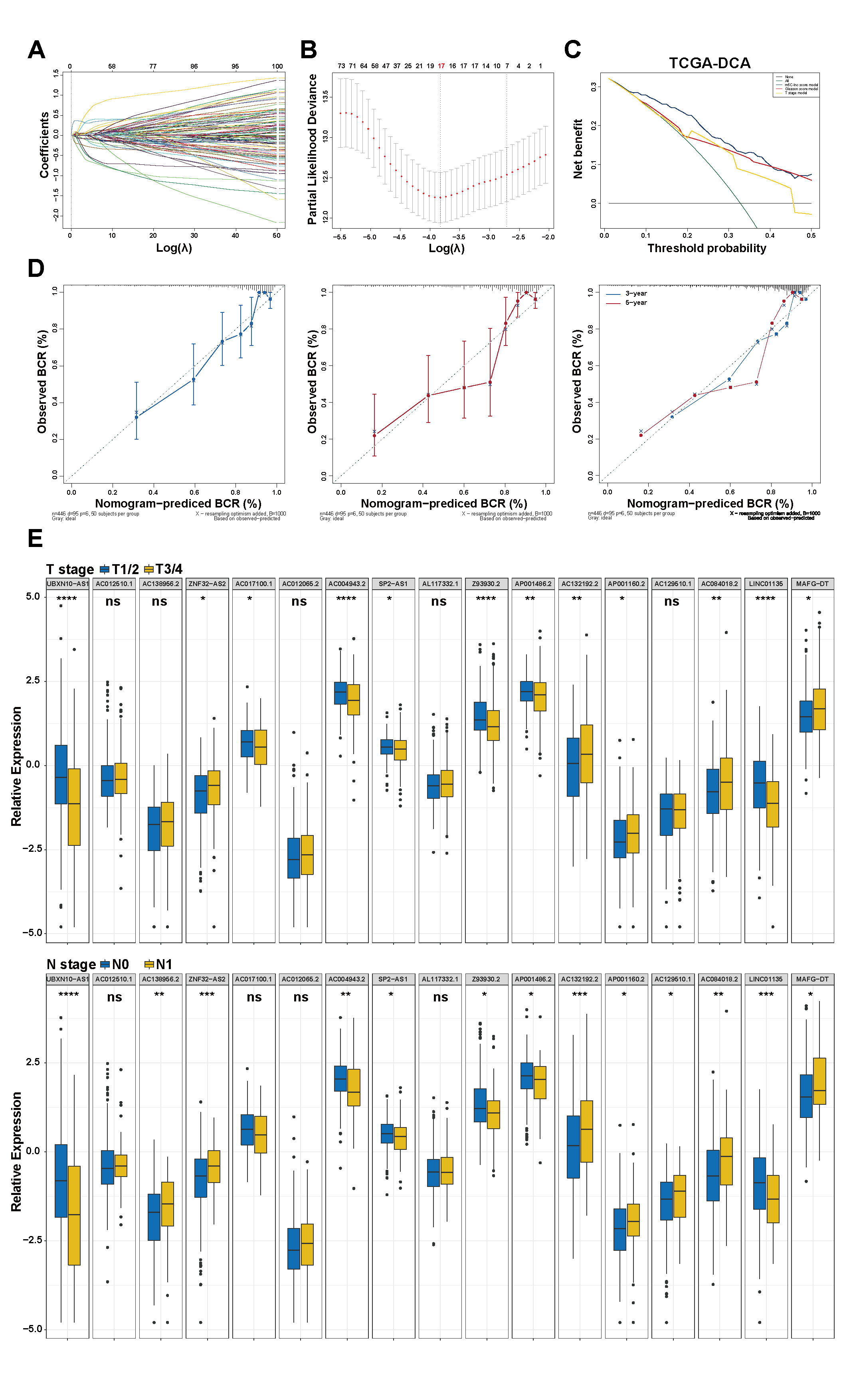

Supplement: Supplementary file 5 [file Image1.JPEG]

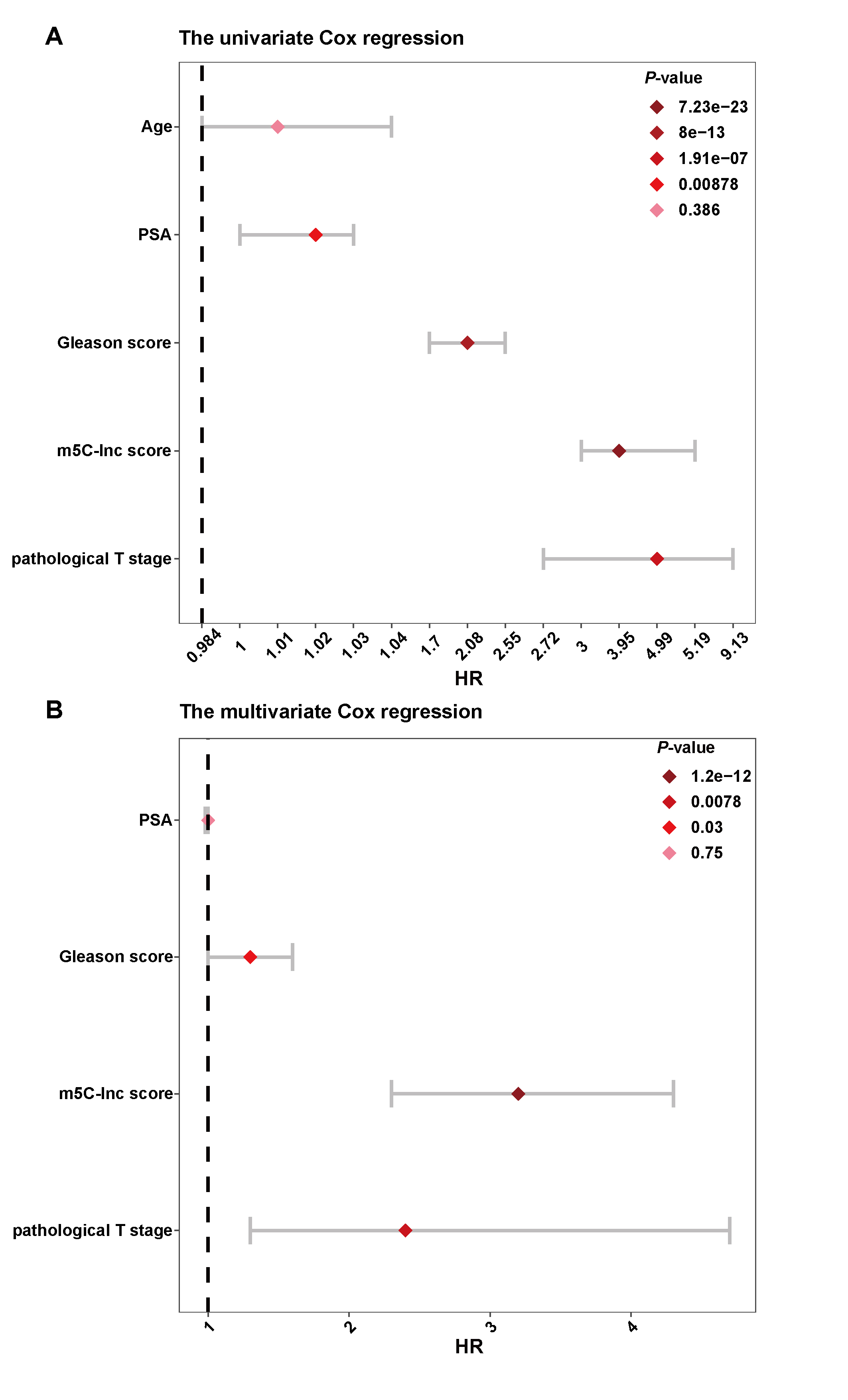

Supplement: Supplementary file 6 [file Image4.JPEG]

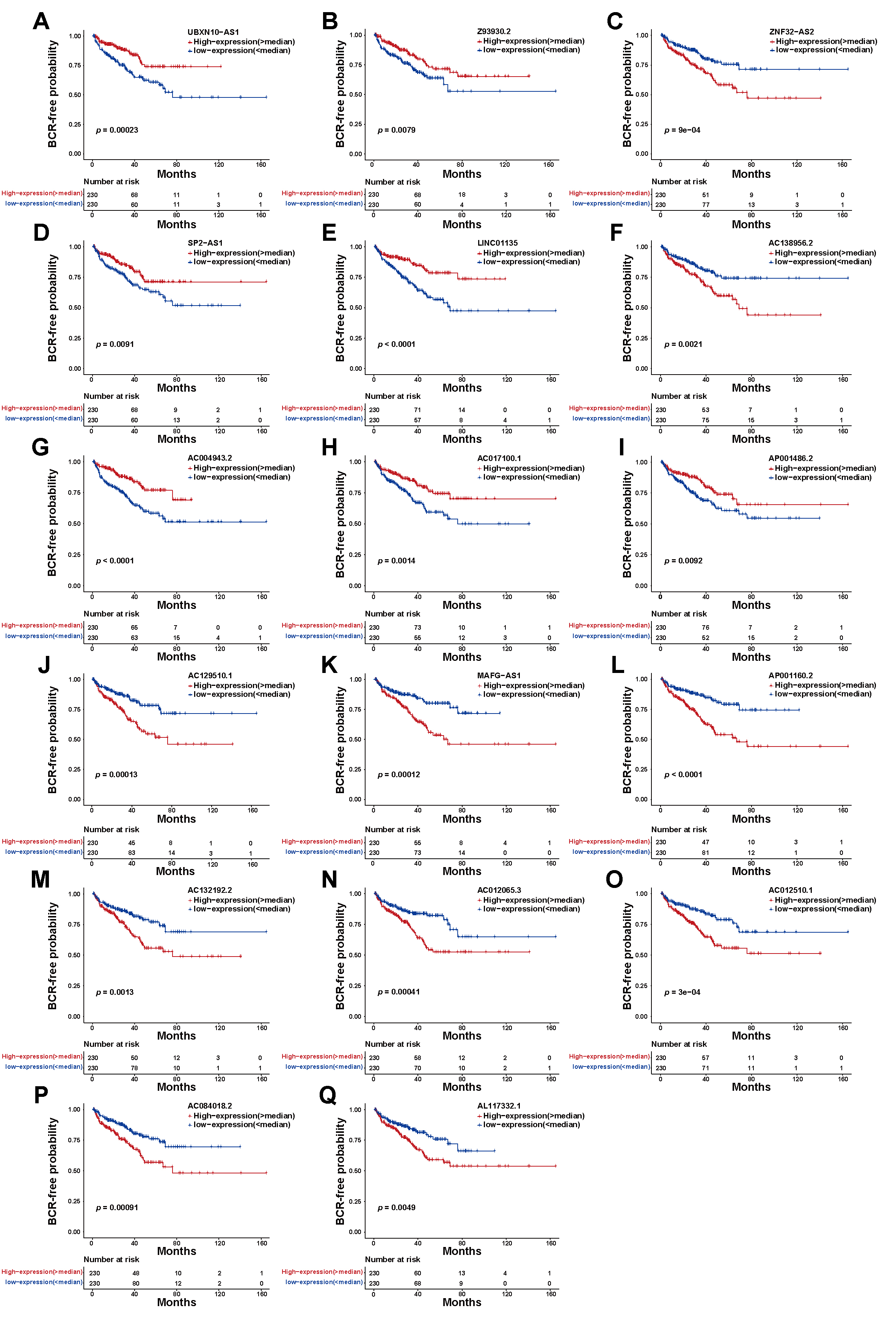

Supplement: Supplementary file 7 [file Image2.JPEG]

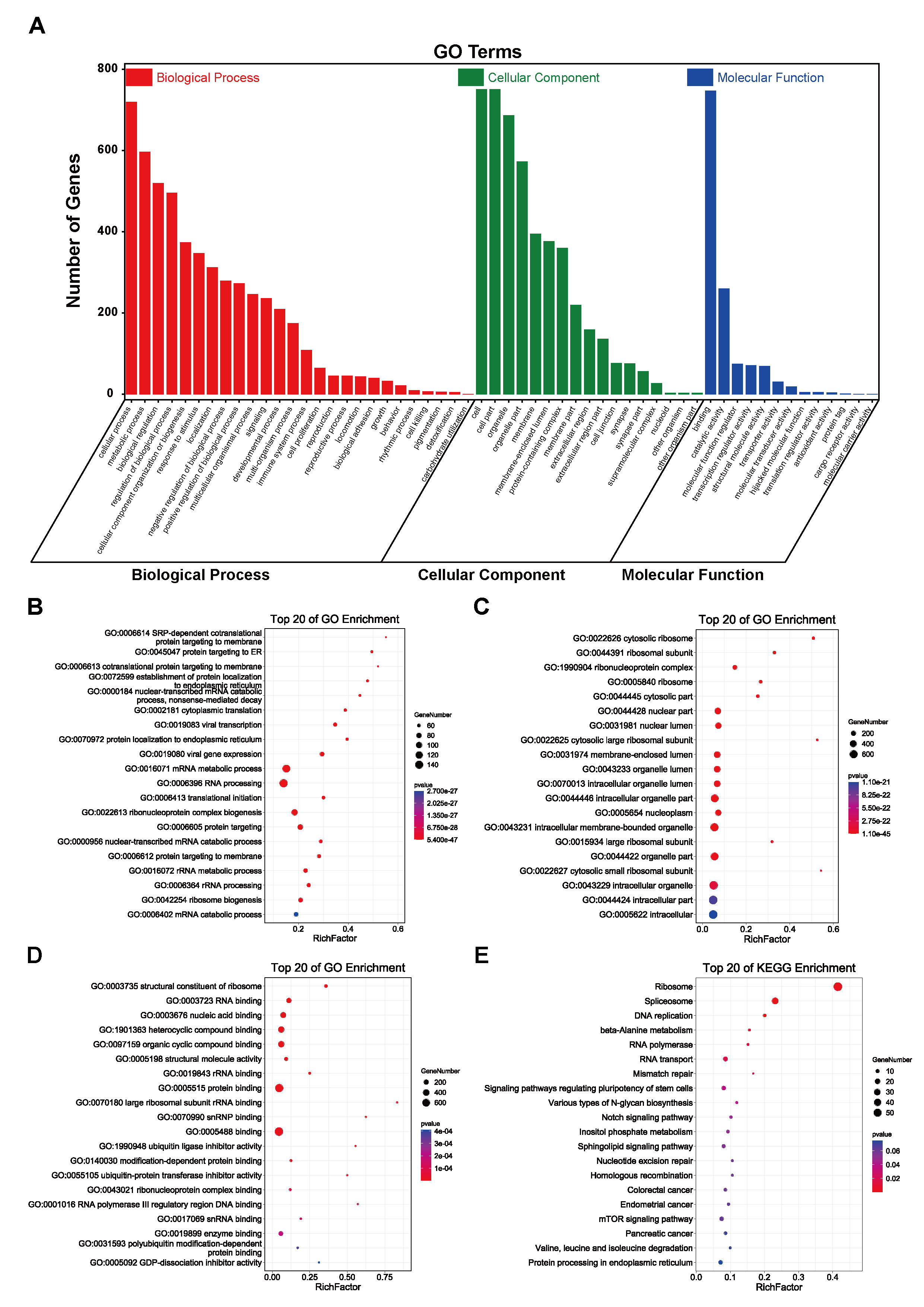

Supplement: Supplementary file 8 [file Image5.JPEG]
