# Supplementary material for: 5-Methylcytosine RNA Methyltransferases-Related Long Non-coding RNA to Develop and Validate Biochemical Recurrence Signature in Prostate Cancer
Source: Front Mol Biosci. 2021 Dec 1;8:775304. doi: 10.3389/fmolb.2021.775304 (PMC8672116; doi:10.3389/fmolb.2021.775304)
Supplement: Supplementary file 4 [file Table8.DOCX]

Table S8 The relation between *MAFG-AS1*’s expression with clinical characteristics in patients with prostate cancer

|  | *MAFG-AS1*’s expression | | |
| --- | --- | --- | --- |
| Variables | n | Mean ± SD | *p* |
| Preoperative PSA (ng/mL) |  |  |  |
| ≤4 | 55 | 1.81±0.82 | 0.164 |
| >4 | 391 | 1.63±0.89 |  |
| Gleason score |  |  |  |
| 6 | 44 | 1.29±0.70 | ＜0.001 |
| 7 | 227 | 1.58±0.84 |  |
| ≥8 | 189 | 1.84±0.92 |  |
| Pathological T stage |  |  |  |
| ≤T2c | 176 | 1.52±0.81 | 0.005 |
| T3+T4 | 279 | 1.75±0.92 |  |
| Lymph node metastasis |  |  |  |
| Negative | 321 | 1.62±0.87 | 0.018 |
| Positive | 74 | 1.89±0.99 |  |
| Surgical margin status |  |  |  |
| R0 | 290 | 1.62±0.87 | 0.145 |
| R1/R2 | 143 | 1.75±0.91 |  |
| BCR |  |  |  |
| Negative | 361 | 1.58±0.86 | ＜0.001 |
| Positive | 99 | 1.94±0.91 |  |
| BCR, biochemical recurrence; PSA, prostatic specific antigen; SD, standard deviation. | | | |

In order to clarify the relationship between *MAFG-AS1* expression level and various clinical characteristics in patients with prostate cancer (PCa), we made a comprehensive analysis via the TCGA-PRAD dataset. Detailed clinical characteristics and grouping were shown in the table above. Firstly, there was no association between *MAFG-AS1* expression level and preoperative PSA levels (PSA≤4: 1.81±0.82, PSA>4: 1.63±0.89, *p*=0.164). Subsequently, patients were divided into three groups based on Gleason score (GS=6, GS=7 and GS≥8), and we found that patients with high Gleason score tended to have high *MAFG-AS1* expression level (1.29±0.70, 1.58±0.84, and 1.84±0.92 respectively, *p*<0.001). Meanwhile, the significant difference was observed in the pathological T stage category (≤T2c: 1.52±0.81, T3/T4: 1.75±0.92, *p*=0.005) but not in the surgical margin status category (R0: 1.62±0.87, R1/R2: 1.75±0.91, *p*=0.145). In addition, the *MAFG-AS1* expression of patients with lymph node metastasis was higher than that of patients without lymph node metastasis (positive: 1.89±0.99, negative: 1.62±0.87, *p*=0.018), and it was consistent with our assumption that *MAFG-S1* expression was higher in patients with BCR (positive: 1.94±0.91, negative: 1.58±0.86, *p*<0.001).
